# Effects of Soil Type on Trace Element Absorption and Fruit Quality of Pepper

**DOI:** 10.3389/fpls.2021.698796

**Published:** 2021-06-30

**Authors:** Zhoubin Liu, Yu Huang, Fangjun Tan, Wenchao Chen, Lijun Ou

**Affiliations:** ^1^College of Horticulture, Hunan Agricultural University, Changsha, China; ^2^ERC for Germplasm Innovation and New Variety Breeding of Horticultural Crops, Changsha, China; ^3^Key Laboratory of Vegetable Biology of Hunan Province, Changsha, China; ^4^Vegetable Institution of Hunan Academy of Agricultural Science, Changsha, China

**Keywords:** pepper, soil, trace element, fruit quality, nutritional quality

## Abstract

The inbred “SJ11-3” pepper was cultured in yellow brown soil, paddy soil, fluvo-aquic soil, and pastoral soil, and the factors affecting the absorption of trace elements and fruit quality were analyzed. The results showed that the physicochemical properties of the soils were significantly different, which led to differences in the nutritional quality of pepper fruits. The pH value had a significant effect on the absorption of trace elements in pepper. The increase of pH promoted the absorption of magnesium and molybdenum but inhibited the absorption of zinc, copper, manganese, and iron. The stepwise multivariable regression analysis showed that the amount of molybdenum in soil was the main factor affecting the total amino acid content of pepper. Total nitrogen, zinc, and copper were the main factors that contributed to the soluble sugar content of pepper, and the available potassium was the major determinant of the vitamin C content of pepper. This study provides new insight on the pepper fruit quality grown on different types of soil with varying levels of trace elements.

## Introduction

Pepper is one of the main vegetable crops in China with an annual planting area of 1.33 million hm^2^. The total economic output is more than 70 billion CNY per year, illustrating its economic significance in many provinces and cities in China (Ma, 2011). With the improvement of living standards, the nutritional value of peppers has received more and more attention. While fruit quality is a complex trait composed of many factors, the absorption, accumulation, and metabolic transformation of soluble solid nutrients, sugars, acids, vitamins, and some special substances during fruit development are important factors determining the flavor and nutritional quality as well as its commercial value. It is well known that the nutritional value of pepper is mainly determined by genetic factors and as well as the environment. As an important agro-ecological factor, the changes in the soil (pH, fertilizer efficiency, enzyme activity, and soil microbe) have an important impact on the quality of pepper.

Recently, research on the nutritional value of crops under different soil conditions (roots) has received increasing attention (Jansen et al., 2012; He et al., 2019). Bao et al. (2006) investigated the soil nutrient status of Newhall navel orange orchard in Hubei Province and its effect on fruit quality. They found a significant linear correlation between soluble solid content and available P and available K content. Base application of selenium fertilizer can increase some essential amino acid content and total amino acid content in the forage and grain of buckwheat (Li et al., 2012). Zhang et al. (2015) demonstrated that nitrogen, phosphorus, and potassium combined with organic fertilizer could increase the content of vitamin C, soluble solids, soluble protein, and soluble sugar in tomato and augment yield. Moreover, Liu et al. (2012) reported that the soluble solid of kiwi fruit was negatively correlated with soil available magnesium and boron, soluble sugar was negatively correlated with soil available magnesium and manganese, and titratable acid was negatively correlated with soil organic matter. In addition, Kong et al. (2013) indicated that organic matter did not contribute to the synthesis of soluble solids and total amino acids. These findings suggest that the impact of soil on crop quality is highly complicated, and the higher efficacy of the fertilizer does not necessarily lead to improved quality.

At present, the traditional cultivation of peppers is mainly based on the theory of “more water and more fertilizer.” Its practice probably can deteriorate the soil structure, reduce the function, waste resource, and cause water pollution and other issues that affect the nutritional quality and safety of pepper. Therefore, in this study, the relationship between different soil conditions and the quality of pepper was investigated by examining the physical and chemical properties, nutrient content, soil microbial and enzyme activities, the nutrient quality, and mineral nutrient absorption of different soil types. These results provide guidance for the improvement of pepper planting soil, proper fertilization, and the enhancement of the quality of pepper.

## Materials and Methods

### Materials and Treatments

This experiment is based on the inbred line “SJ11-3” provided by the Hunan Provincial Vegetable Research Institute. It has long, spirally shaped fruit, good taste, and high vitamin C content, which is widely used in the breeding of screw pepper. It was planted from April to September 2016 in the yellow brown soil (Xiangyin County, 112°52' E, 28°14' N), paddy soil (Ningxiang County, 112°33' E, 28°16' N), pastoral soil (Changsha County, 113°4' E, 28°14' N), and fluvo-aquic soil (Changde City, 111°41' E, 29°1' N), and each soil was divided into three plots. The four soil types are distributed in the central and northern parts of Hunan Province with little difference in geographical location: longitude and latitude. Therefore, the temperature change, light intensity, and precipitation during the planting period were generally consistent, and there was no significant climate difference.^1^ The tested pepper seeds were seeded in the plug tray after germination, and the seedlings with strong and consistent growth were obtained in the five-leaf period. For transplantation, 30 peppers were planted in each plot, and the row spacing × plant spacing was 40 cm × 40 cm. All the plants were planted in open field, and fertilization and water management were consistent during planting. After planting, the pepper green ripe fruits were obtained at 35 days after flowering, and 20 peppers were taken from each plot. Meanwhile, nine plants with picked peppers were selected and 200 g soil was taken from the roots of 0–15 cm deep of each plant. The obtained peppers and soil were frozen at −80°C by liquid nitrogen and used for the following experiments.

### Determination of Physical and Chemical Properties

The pH value of soil was determined by potentiometric electrode method. The bulk density was quantified by the ring knife method. The contents of zinc, copper, manganese, and molybdenum in soil and pepper fruit were measured by inductively coupled plasma mass spectrometry method. The total nitrogen content in soil was determined by semi-micro Kay method. The content of nitrogen was examined by the alkali diffusion method. The content of total phosphorus was measured by HClO_4_-H_2_SO_4_ digestion molybdenum ruthenium colorimetric method. The content of available phosphorus was calculated by sodium bicarbonate extraction-molybdenum ruthenium colorimetry. The total potassium content was determined by H_2_SO_4_-H_2_O_2_ digestion-flame photometric method. The available potassium content was quantified by ammonium acetate extraction-flame photometry.

The urease activity of soil was determined by sodium phenolate-sodium hypochlorite colorimetric method. The phosphatase activity was measured by the phenyl phosphonate colorimetric method. The invertase activity was quantified by 3,5-dinitrosalicylic acid colorimetric method. The catalase activity was examined by potassium permanganate titration. The enzyme activities are expressed as the amount of substance produced by the unit weight of air-dried soil during the culture period.

The soluble sugar of pepper fruit was determined by the anthrone colorimetric method. The total amino acid, vitamin E, and vitamin C were quantified by kit (Nanjing Institute of Bioengineering).

### Analysis of Soil Microbial Diversity

#### Extraction of Total Soil Microbial DNA

The total soil microbial DNA was extracted with Soil DNA Extraction Kit (Stamford, CT, United States). The integrity of DNA was verified with 1% agarose gel electrophoresis. The concentration and purity of the extracted DNA were measured with Mini-Drop (Thermo Fisher Scientific, Waltham, MA, United States).

#### PCR Amplification and Sequencing

The extracted total DNA was used as the template for PCR amplification. Primers targeting V3 + V4 regions of bacterial 16S rDNA gene (F:5'-ACTCCTAC GGGAGGCAGCA-3'; R: 5'-GGACTACHVGGGTWTCTAAT-3') and primers targeting fungal ITS1 region (F: 5'-CTTGGTCATTTAGAGGAAGTAA-3'; R: 5'-GCTGCGT TCTTCATCGATGC-3') were designed by Biomarker Science and Technology Service (Beijing, China), and V3 + V4 regions and ITS1 region were amplified with PCR, respectively. The PCR reaction system was as follows: pre-denaturation at 95°C for 5 min, followed by 25 cycles of denaturation at 95°C for 30 s, annealing at 50°C for 30 s, extension at 72°C for 40 s, and the final extension at 72°C for 7 min. The amplified DNA products were purified, quantified, and uniformed to form the sequencing library. The PCR-amplified DNA products were sequenced using Illumina MiSeq 2500 by Biomarker Science and Technology Service (Beijing, China).

#### DNA Sequencing Analysis

(1) Merge of PE Reads: The merger of the original sequences obtained was conducted with FLASH v1.2.7 software. According to the minimum overlap length of 10 bp, the maximal mismatched ratio that was allowed in the overlapping region was 0.2. Merge of reads of each sample was conducted, and the merger sequences were obtained, i.e., the original Tags data (Raw Tags). (2) Filtration of Tags: the Raw Tags obtained by merger were filtrated with Trimmomatic v0.33. The parameter was set as the 50 bp window. If the mean quantity value was lower than 20, the back-end nucleotide bases starting from the window were removed. After filtration and quantity control, the length of DNA fragments was shorter than 75% of the Tags. The high-quality Tags (Clean Tags) were obtained. (3) Removal of Chimera: the chimera sequences were identified and removed with UCHIME v4.2 software. Finally, the effective tags were obtained. UCLUST in QIIME (version 1.8.0) was used to conduct cluster analysis of Tags at the level of 97% similarity. OTU and RDP classification for all the sequences was conducted to obtain OTUs based on the bacterial and fungal taxonomic databases, and the numbers of OTU were calculated for statistical tests.

### Data Analysis

In this study, the results were expressed as “mean ± standard error,” and data analysis was performed using Excel 2010 and SPSS 17.0. The significance of multiple comparisons was determined by Duncan’s test.

## Results

### Differences in Physical and Chemical Properties of Different Soils

According to the soil pH and bulk weight grading standards, we found a significant difference in pH among the four types of soil: yellow brown soil, paddy soil, fluvo-aquic soil, and pastoral soil (*p* < 0.05), and the range was 4.20–6.42, all of which were acidic soils (Table 1). The paddy soil was strongly acidic, with the lowest pH. On the contrary, the pastoral soil was slightly acidic, and the pH value was significantly higher than that of the other three types (*p* < 0.05). Also, the overall bulk density of the four soils is relatively high, considered as compact or semi-compact. The fluvo-aquic soil has the highest compactness, with no significant difference with the paddy soil, but is 11.53% higher than the yellow brown soil with the lowest bulk density.

**Table 1:** Soil acidity and bulk density grading.

Soils	pH	pH grading	Bulk density/g/cm^3^	Compactness
Yellow brown soil	4.95b	Acidic	1.30c	Partial tight
Paddy soil	4.20c	Strong acidic	1.44a	Tight
Fluvo-aquic soil	4.82b	Acidic	1.45a	Tight
Pastoral soil	6.42a	Slightly acidic	1.35b	Partial tight

*Each value is the mean* ± *standard deviation (SD, n = 6). The effects are significant at p < 0.05 with a one-way ANOVA. Different lowercase letters represent significant differences*.

### Comparison of Nutrient Contents in Different Soils

The fertility of rhizosphere is significantly dependent on the soil type (Table 2). The content of total nitrogen and alkali nitrogen was the highest in paddy soil, which was 99.37 and 66.49% higher than that in the lowest pastoral soil. The total phosphorus and available phosphorus were the highest in fluvo-aquic soil, followed by pastoral soil, and the lowest in yellow brown soil, which were only 41.26 and 7.08% of the fluvo-aquic soil, respectively. The highest content of total potassium was detected in the yellow brown soil, which was significantly higher than that in the other soils. There was no significant difference in the content of total potassium among paddy soil, fluvo-aquic soil, and pastoral soil. The available potassium content was the highest in paddy soil, followed by fluvo-aquic soil and pastoral soil. The content of available potassium was the lowest in yellow brown soil, which is only 19.82% of that in the paddy soil.

**Table 2:** Analysis of nutrient contents in different soils.

	Total nitrogen g/kg	Total phosphorus mg/kg	Total potassium mg/kg	Alkaline hydrolyzed nitrogen mg/kg	Available phosphorus mg/kg	Available potassium mg/kg
Yellow brown soil	2.60 ± 0.22b	302.61 ± 7.99d	8.50 ± 0.17a	90.77 ± 4.45bc	11.36 ± 0.56c	13.23 ± 1.52d
Paddy soil	3.17 ± 0.30a	453.84 ± 19.81c	7.35 ± 0.23b	128.53 ± 8.94a	60.49 ± 5.27b	66.74 ± 4.81a
Fluvo-aquic soil	1.97 ± 0.19bc	733.34 ± 18.03a	7.95 ± 0.25b	107.38 ± 3.22b	160.44 ± 6.33a	45.31 ± 2.47b
Pastoral soil	1.59 ± 0.16c	587.50 ± 34.43b	7.58 ± 0.16b	77.20 ± 5.32c	65.37 ± 6.25b	23.76 ± 1.35c

*Each value is the mean ± standard deviation (SD, n = 6). The effects are significant at p < 0.05 with a one-way ANOVA*. Different lowercase letters represent significant differences.

### Comparison Enzyme Activities in Different Soils

**Figure 1:**
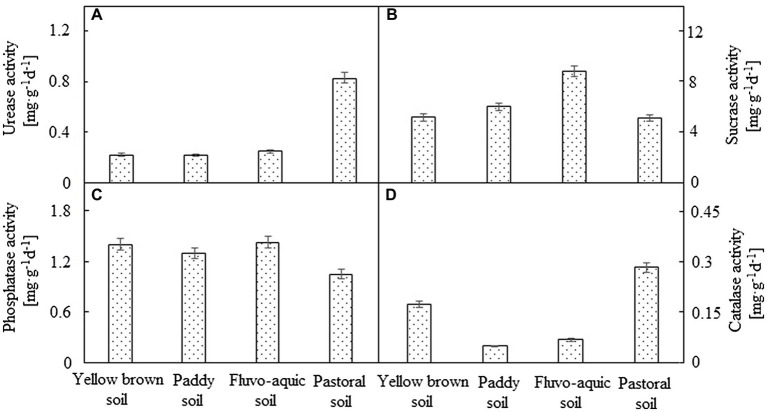
The enzyme activities of different soils. **(A–D)** represented urease, sucrase, phosphatase and catalase activity, respectively.

There were significant differences in enzyme activities among the four soils (Figure 1). The soil urease activity was the highest in pastoral soil, which was significantly higher than the other soils (*p* < 0.05). The urease activity was the lowest in paddy soil, which was only 26.01% of that in pastoral soil. No significant difference in urease activity was observed between yellow brown soil and fluvo-aquic soil (*p* < 0.05). Fluvo-aquic soil exhibited the highest activity of sucrase among the four types of soil, which was 36.19% higher than that of pastoral soil, which showed the lowest activity of sucrase. Soil phosphatase displayed the highest activity in fluvo-aquic soil and yellow brown soil, followed by paddy soil, and no significant difference was detected among the three (*p* < 0.05), with the lowest phosphatase activity in the pastoral soil. However, catalase activity was the highest in the pastoral soil, followed by yellow brown soil, and the lowest in the paddy soil, which was only 17.23% of the activity in the pastoral soil.

### Comparison of Nutrient Composition of Pepper Grown in Different Soils

**Figure 2:**
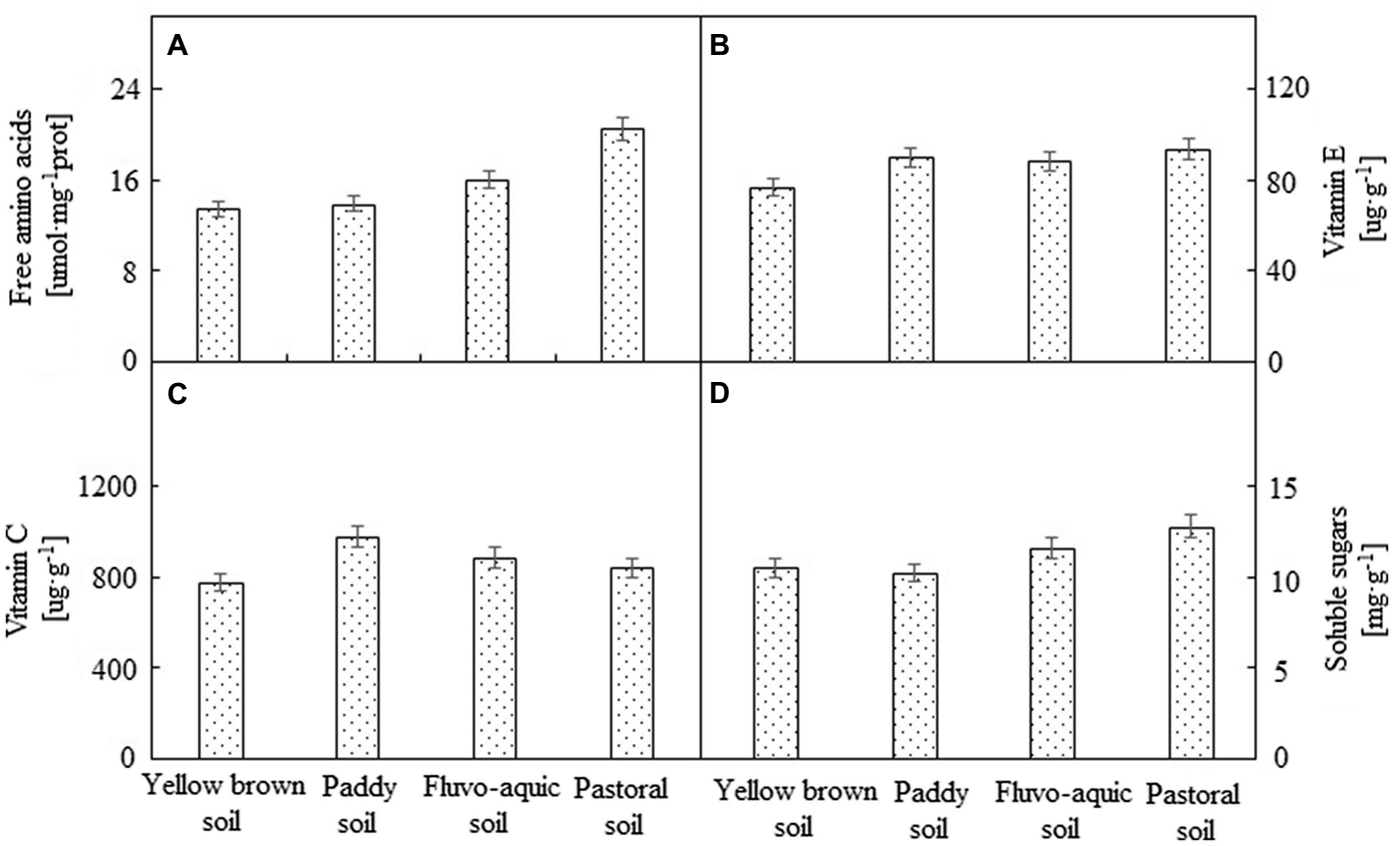
The nutrient composition of pepper grown in different soils. **(A–D)** represented Free amino acid, vitamin E,vitamin C and soluble sugars, respectively.

The nutrient composition of pepper varied due to the type of soil (Figure 2). The total amino acid content of pepper grown in the pastoral soil was significantly higher than that of the other three soils, which was 28.28% higher than that of the second highest fluvo-aquic soil. No significant difference in amino acid content (*p* < 0.05) was observed among the peppers grown in fluvo-aquic soil, paddy soil, and yellow brown soil. The content of soluble sugar was the highest in the pepper grown in the pastoral soil, followed by the pepper planted in the fluvo-aquic soil. There was no significant difference between the two (*p* < 0.05). The content of soluble sugar was the lowest in the pepper grown in the paddy soil, which was 19.92% lower than that in the pastoral soil. The highest vitamin E content was determined in the pepper grown in the pastoral soil, and there was no significant difference among the other three soils (*p* < 0.05). The content of vitamin C was the highest in the paddy soil, followed by the fluvo-aquic soil and pastoral soil. The pepper planted in the yellow brown soil showed the lowest vitamin C content, which was only 79.68% of the paddy soil.

### Effects of Trace Element Contents in Different Soils on the Absorption of Trace Elements in Pepper

The contents of trace elements zinc, copper, magnesium, manganese, molybdenum, and iron were significantly different among different soil types and pepper fruits (Table 3). The content of zinc in the four types of soils was significantly different (*p* < 0.05), among which the content of pastoral soil was the highest, and the content of yellow brown soil was the lowest, which was 28.85% lower than that of pastoral soil. The content of zinc in pepper was the highest in the pastoral and paddy soils, which is significantly higher than that in pepper planted in the fluvo-aquic and yellow brown soil (*p* < 0.05). The content of copper was the highest content in the pastoral soil and the lowest in the yellow brown and fluvo-aquic soils, and there was no significant difference between the two (*p* < 0.05). The copper content in the pepper was the highest in the paddy soil, followed by the yellow brown soil, while the amount of copper in pepper grown in the pastoral soil and fluvo-aquic soil was the lowest. The content of magnesium in fluvo-aquic soil and yellow brown soil was the highest and was the lowest in paddy soil, which was only 61.48% of that of pastoral soil. The magnesium in pepper was the highest in fluvo-aquic soil, but it is not significantly different from that in yellow brown soil and pastoral soil (*p* < 0.05). The content of manganese in the soil was the highest in the pastoral soil and fluvo-aquic soil, and the lowest in the paddy soil, which was significantly lower than that in all the other soils (*p* < 0.05). In the pepper, the content of manganese in the paddy soil was the highest, which was 172.56% higher than that in the lowest yellow brown soil. The content of molybdenum was the highest in pastoral soil, which was significantly higher than all the other soils (*p* < 0.05), and the lowest in fluvo-aquic soil and yellow brown soil,; there was no significant difference between them (*p* < 0.05). The content of molybdenum was the highest in peppers planted in the pastoral soil, while the peppers planted in the yellow brown soil were the lowest, which was only 17.40% of that in the pastoral soil. The content of iron in the pastoral soil was the highest, which was significantly higher than that in the other soils. The content of iron in pepper was the highest in the paddy soil, which was significantly higher than that in the other soils (*p* < 0.05).

**Table 3:** Effects of different soil trace elements on the absorption of trace elements in pepper.

		Yellow brown soil	Paddy soil	Fluvo-aquic soil	Pastoral soil
Zinc	Pepper (mg/kg)	3.07 ± 0.19b	5.14 ± 0.12a	3.25 ± 0.11b	5.18 ± 0.21a
	Soil (mg/kg)	108.13 ± 3.22d	123.26 ± 2.51b	115.41 ± 1.09c	151.98 ± 4.89a
Copper	Pepper (mg/kg)	1.12 ± 0.08b	1.47 ± 0.15a	0.75 ± 0.11c	0.76 ± 0.04c
	Soil (mg/kg)	16.85 ± 0.86c	25.39 ± 1.02b	15.77 ± 0.63c	43.60 ± 1.26a
Magnesium	Pepper (g/kg)	0.11 ± 0.01a	0.07 ± 0.01b	0.12 ± 0.02a	0.10 ± 0.01a
	Soil (g/kg)	5.07 ± 0.26a	3.24 ± 0.19c	5.27 ± 0.34a	3.75 ± 0.22b
Manganese	Pepper (mg/kg)	1.64 ± 0.09d	4.47 ± 0.17a	2.39 ± 0.23bc	2.02 ± 0.19c
	Soil (mg/kg)	347.10 ± 12.88b	143.41 ± 7.57c	483.20 ± 18.03a	507.37 ± 25.42a
Molybdenum	Pepper (μg/kg)	4.71 ± 0.11d	7.92 ± 0.23c	9.15 ± 0.17b	27.03 ± 0.98a
	Soil (mg/kg)	1.60 ± 0.07c	2.52 ± 0.09b	1.74 ± 0.06c	3.02 ± 0.13a
Ferrum	Pepper (mg/kg)	15.41 ± 0.14c	27.44 ± 0.33a	15.93 ± 0.17c	20.25 ± 0.27b
	Soil (g/kg)	3.37 ± 0.19b	3.25 ± 0.09b	3.21 ± 0.11b	4.12 ± 0.22a

*Each value is the mean ± standard deviation (SD, n = 6). The effects are significant at p < 0.05 with a one-way ANOVA. Different lowercase letters represent significant differences*.

### Analysis of Microbial Diversity in Different Soils

**Figure 3:**
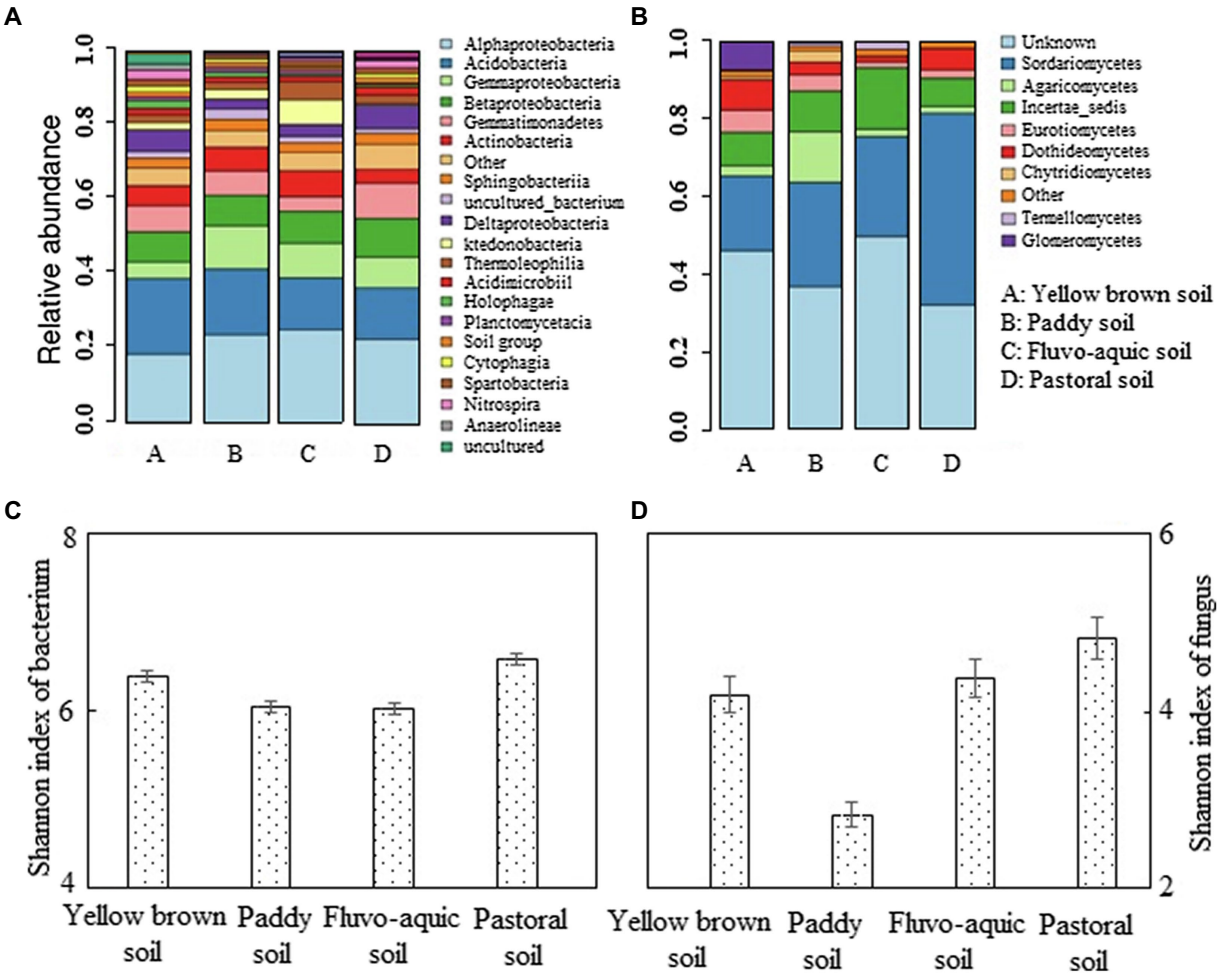
Analysis of bacterial and fungal levels in different soils. **(A,B)** represented the abundance of bacterium and fungus, respectively. **(C,D)** represented the shannon index of bacterium and fungus, respectively.

The microbial diversity was high in different soils, and the number of bacterial species of different soil types was significantly higher than the fungal species (Figures 3A,B). The bacteria in the pepper planting soil were mainly *Alphaproteobacteria, Acidobacteria, Gammaproteobacteria, Betaproteobacteria, Gemmatimonadetes*, and *Actinobacteria*, which accounted for more than 60% in number. Among them, α-proteobacteria, acid bacillus, and β-proteobacteria were mostly identical in number, while γ-proteobacteria was the most diverse in paddy soil and the least diverse in yellow brown soils. *Gemmatimonadetes* were the most enriched in pastoral soil, while is the lowest in number in fluvo-aquic soil. *Actinomycetes* were the most abundant in paddy soil and fluvo-aquic soil, and the rarest in pastoral soil. The Shannon index of bacterial community in Pastoral soil and yellow brown soil was 6.58 and 6.39, which were significantly higher than those in paddy soil and fluvo-aquic soil (*p* < 0.05; Figure 3C). The differences in fungi species in pepper-planted soils were more evident. The fungi in the four soils were mainly unknown fungi, *Sordariomycetes, Agaricomycetes, and Incertae sedis*, with unknown fungi and *Sordariomycetes* accounting for 60–80% of the total fungal species in the four soils. Among them, the species of *Sordariomycetes* were the most abundant in the pastoral soil, and the lowest in the yellow brown soil. The species of the *Agaricomycetes* were the most abundant in the paddy soil, which was significantly higher than the other three soils. The *incertae sedis* was the most dominant in the fluvo-aquic soil, followed by the paddy soil. The Shannon index of the fungal community in the four regions was also significantly different (*p* < 0.05; Figure 3D). The Shannon index of fungal community in pastoral soil was the highest, which was 4.83. The Shannon index of paddy soil fungus community was the lowest, which was 41.40% lower than that of pastoral soil.

### Multiple Regression Analysis of Nutrients and Soil Factors in Pepper

Since the nutrient level of the soil will change plant growth, development, and metabolism, and the soil composition is a complex system, it is imperative to transform the measured factors to eliminate the nonlinearity in the linear regression analysis. Therefore, soil factors, including pH (X1), bulk density (X2), bacterial Shannon index (X3), fungal Shannon index (X4), total nitrogen (X5), total phosphorus (X6), total potassium (X7), alkaline hydrolyzed nitrogen (X8), available phosphorus (X9), available potassium (X10), urease (X11), invertase (X12), phosphatase (X13), catalase (X14), zinc (X15), copper (X16), magnesium (X17), manganese (X18), molybdenum (X19), and iron (X20), were treated as independent variables, and the total amino acid content (Y1), soluble sugar (Y2), vitamin E (Y3), and vitamin C (Y4) of pepper were considered as the dependent variables. We eliminated the least influential factor in the objective function by stepwise multiple regression. The stepwise multiple regression function between the nutrient composition of the pepper and the main soil factors was determined as follows: Y1 = 12.145 + 0.974 × X19, R2 = 0.949; Y2 = 13.784–0.981 × X5 + 0.301 × X15 + 0.013 × X16, R2 = 0.908; and Y4 = 786.235 + 0.876 × X10 + 0.154 × X15–0.058 × X7, R2 = 0.984, while the valid function for Y3 was not obtained. These results showed that the main factor affecting the total amino acid content of pepper was the content of molybdenum in soil, the main factors determining the content of soluble sugar were the contents of total nitrogen, zinc, and copper in soil, and the main factor controlling the content of vitamin C is the contents of available potassium, zinc, and total potassium in soil.

## Discussion

Zinc, copper, magnesium, manganese, molybdenum, and iron are heavy metal elements and trace elements that play an important role in the growth and development of living organisms. pH is one of the most important factors affecting the absorption and distribution of heavy metals (Anderson and Rubin, 1982). Studies have shown that elevated pH prompts the increase of rhizosphere copper (Lexmond and Vorm, 1981; Chaignon et al., 2002), while leads to a decrease in copper in plants (Li, 2005). Li et al. (2003) found that the application of acidic rhizosphere fertilizer reduced the soil pH within 2 cm of the fertilization area, which increased the effective iron content in the area and promoted the absorption of iron by plants. In this experiment, the content of copper and iron in the pastoral soil was the highest, but the content of copper and iron in the pepper was at a low level, whose level in the paddy soil was the highest. This was because the pH value of the pastoral soil is significantly higher than the other soils, causing the copper and iron to be enriched in the soil. However, this enrichment may be mainly in the form of copper or ferrous salts, rather than the ions which are easily absorbed by plants. This is consistent with previous studies on the adsorption of copper and iron in soil (Wallace et al., 1980; Xu et al., 2005). Previous studies have found that the availability of zinc and manganese in the rhizosphere and the absorption of zinc and manganese by plants are negatively correlated with soil pH (Eriksson, 1989; He and Singh, 1994; Zhang et al., 2012). Fu et al. (2013) examined the soil in Bijie tobacco region and demonstrated that the effective Mn and effective Zn in soil were the highest when pH <5. In the current experiments, the amount of zinc and manganese in other soils was significantly lower than that in the paddy soil. Also, no significant difference was observed between the zinc content in the peppers grown in the paddy soil and pastoral soil, while the manganese content was significantly higher in the paddy soil. This result could be resulted from the higher pH of the pastoral soil, which enhanced the adsorption of soil to Zn^2+^ and Mn^2+^, and reduced the concentration of these ions in the soil. Moreover, it decreased the available zinc and manganese which are conducive to the absorption of plants and vice versa in the paddy soil with a low pH. In general, the absorption of molybdenum and magnesium by plants is positively correlated with the content of the elements in the soil, but the molybdate is barely soluble in acidic soils (Shen, 2010). However, the exchangeable magnesium is enhanced with the increasing soil acidity (Diao et al., 2013). In this experiment, the relatively high pH of the pastoral soil promoted the activity of available molybdenum and exchangeable magnesium in the soil and prompted the absorption of molybdenum and magnesium by the pepper, which resulted in the molybdenum element of the plant being significantly higher than the other three soils. The content of magnesium was not significantly different from the yellow brown soil and fluvo-aquic soil with high magnesium level in the soil. Although the paddy soil had higher total molybdenum content, the low soil pH decreased the effective molybdenum content, resulting in lower molybdenum content in the fruit, which was consistent with the results of Ye et al. (2011). Therefore, the current study suggests that elevated soil pH can promote the absorption of magnesium and molybdenum by pepper, and reduced the absorption of zinc, copper, manganese, and iron. It also indicates that pH can affect the absorption of trace elements in pepper by regulating their availability in the soil.

Amino acids, soluble sugars, vitamin E, and vitamin C are important determinants of pepper quality, whose contents determine not only the nutritional but also the commercial value of peppers. Studies have demonstrated that the total amino acid in plants is positively correlated with the N, P, and K absorbed (Shi et al., 2019; Wen et al., 2020; Zhao et al., 2020). In this experiment, the content of nutrients in paddy soil and fluvo-aquic soil was higher, but the total amino acid of the planted pepper was not significantly different from that of yellow brown soil with lower soil nutrient, but significantly lower than that of other soils with lower nutrient level. This indicates that in addition to the nutrient-related accumulation of total amino acids, other factors are regulating their accumulation in plants. Yang and Jiang (2016) reported that the high soil sucrase and phosphatase activities inhibited the accumulation of total ginsenosides, total water-soluble proteins, and total amino acids. Nie et al. (2008) investigated pakchoi and found that the application of molybdenum significantly increased the content of some essential amino acids and non-essential amino acids. In this study, the stepwise multivariable regression analysis indicated that the content of molybdenum in soil is the most important factor that determines the total amino acid content of pepper, which is consistent with the results of previous studies in soybean (Liu and Yang, 2003). It suggests that the effects of other factors, such as invertase and phosphatase on the total amino acids of pepper, are far less than those of molybdenum. Changes in stress, soil pH, and trace element content can affect the amount of soluble sugar in plants. Tang et al. (2007) found that zinc fertilizer had a significant effect on the soluble sugar content of pakchoi, whose content first increased and then decreased with the increase of zinc fertilizer application. Also, they demonstrated that zinc significantly boosted the vitamin C content in pakchoi. Zhang et al. (2015) uncovered that the soluble sugar content in tomato was significantly positively correlated with soil urease and catalase. Also, Zhang et al. (2016) reported that the proper application of copper micro-fertilizer significantly increased the soluble sugar, soluble protein, and starch of the bulb of saffron. This result is consistent with our current study that the highest content of zinc and copper is in the pastoral soil and the highest soluble sugar content in the planted pepper. The results of the stepwise regression analysis illustrated that soil nitrogen, zinc, and copper were the main factors affecting the soluble sugar of pepper, while urease and catalase were not, which were rather mainly involved in the transformation of nutrients in the soil, but not directly involved in on the plants.

Soil total nitrogen is the most important factor affecting soluble sugar content. Studies have shown that proper nitrogen application can facilitate the accumulation of soluble sugar, while excessive nitrogen application will lead to a decrease in soluble sugar (Karimi and Koulivand, 2019). The changes in soil nitrogen content can consequently regulate the conversion and transfer of soluble sugar in plants (Sung et al., 2015). In production, attention should be paid to improve the availability of nitrogen and efficacy of its usage, and not blindly increase the nitrogen content in the soil. Potassium is known as the “quality-controlling element,” which promotes fruit enlargement, increases yield, and improves nutritional quality (Pettigrew, 2010). Previous reports have demonstrated that the application of potassium increases vitamin C in fruits (Chen et al., 2011; Ning et al., 2011; Lu et al., 2015). In this study, the content of available potassium in paddy soil was the highest, leading to the highest content of vitamin C in peppers. The stepwise regression showed that available potassium was the most important factor affecting the content of vitamin C in pepper. On the other hand, the total potassium in soil negatively affected the content of vitamin C. This may indicate that it is not the potassium content in the soil that determines the vitamin C content of the pepper, but the content of available potassium in the soil. Therefore, when applying potassium fertilizer, efforts should be made to increase the content of available potassium rather than enhancing the total amount of potassium. In addition to available potassium and total potassium, zinc is also a major factor affecting vitamin C. Studies have indicated that zinc can increase the vitamin C content of fruits in red dates (Kang and Li, 2012) and eggplant (Wang et al., 2011). We did not obtain the formula for vitamin E by stepwise regression, which indicates that vitamin E content is not affected by several major factors, but the overall effects of all soil factors. Zhang et al. (2009) reported that a high level of calcium and phosphorus have an effect on the accumulation of vitamin E in wheat germ callus, and the effect from the phosphorus is much stronger. In the current study, the available phosphorus content of the four types of soils was significantly different from each other. However, the content of vitamin E in the fruit was similar among the four soils. This may be because the amount of effective phosphorus in the soil affects the absorption in the plant with a critical saturation of 30 mg/kg, beyond which the increase of the application of phosphate fertilizer cannot generate further effects (Fan et al., 2005).

Soil microbes are the driving force for the transformation and circulation of soil organic matter and nutrient and play an important role in many aspects of the formation and development of soil fertility. The soil microbial diversity is also closely related to soil function and maintains the dynamic balance of the soil system (Nathalia and Daniel, 2011; Haytham and Sharkawi, 2012). The Shannon-Werner diversity index of bacteria and fungi in the pastoral soil in this study was higher than the other soils, indicating that the microbial population of the pastoral soil is richer and more active. Soil microbes function as an important part of the cyclical transformation of soil C, N, P, and other nutrients, and it also plays a critical role in promoting the energy exchange between plants and soil, which explains the higher nutrient content of pepper grown in the pastoral soil. In addition, the analysis of bacterial diversity in the four soil types demonstrated that the rhizosphere of pepper was enriched with a large number of deformed bacteria. Presently, the enrichment of deformed bacteria has been observed in the rhizosphere of *Arabidopsis thaliana* (Fierer et al., 2007) and maize (Peiffer et al., 2013), indicating that the deformed bacteria can adapt to the rhizosphere microenvironment of various plants. However, results from the current study do not support the hypothesis that soil microbes can directly affect the nutritional quality of pepper. Na et al. (2016) found that the content of total phosphorus and available phosphorus was significantly correlated with the ratio of *Acidobacteria, Sordariomycetes, Basidiomycetes, and Zygomycetes*. This result suggests that changes in soil microbes may indirectly affect crops by changing the nutrient composition and activity of the soil. In summary, many factors, including soil nutrient, pH, enzyme activity, and soil microbe, contribute to the quality of pepper. In the future, to maximize the efficacy of improving the nutrient quality of pepper, more attention should also be paid to the effects of microorganism when adjusting the particular nutrient composition of the soil.

## Conclusion

In this study, the nutritional quality of pepper fruits grown in soil with different physical and chemical properties was different, indicating that there was a certain relationship between pepper quality and soil properties. The results of stepwise multivariable regression analysis showed that the amount of molybdenum in soil is the main factor affecting the total amino acid content of pepper. Total nitrogen, zinc, and copper are the main factors that contribute to the amount of soluble sugar of pepper. Also, available potassium is the major determinant of the vitamin C content of pepper. This study provides a new insight for analyzing the relationship between soil trace elements and the pepper fruit quality.

## Data Availability Statement

The datasets presented in this study can be found in online repositories. The names of the repository/repositories and accession number(s) can be found at: https://www.ncbi.nlm.nih.gov/ (PRJNA577242 and PRJNA577978).

## Author Contributions

ZL: formal analysis and writing—original draft. LO: funding acquisition, project administration, supervision, and writing—review and editing. ZL and YH: investigation. ZL and FT: methodology. WC: resource. All authors have read and approved the final manuscript.

### Conflict of Interest

The authors declare that the research was conducted in the absence of any commercial or financial relationships that could be construed as a potential conflict of interest.

## Footnotes

^1^http://hn.cma.gov.cn/
